# Cytoplasmic nucleophosmin has elevated T199 phosphorylation upon which G2/M phase progression is dependent

**DOI:** 10.1038/srep11777

**Published:** 2015-06-30

**Authors:** Narisa Chan, Tit Meng Lim

**Affiliations:** 1 Department of Biological Sciences, National University of Singapore, 14 Science Drive 4, Singapore 117543.

## Abstract

The cytoplasmic mutant of nucleophosmin (NPMc) is found approximately in one-third of acute myeloid leukemia (AML) cases and is highly associated with normal karyotype. Whereas previous studies have focused on wtNPM in centrosome duplication, we further elucidate the role of NPM in the cell cycle by utilizing the increased cytoplasmic load of NPMc. Overexpression of NPMc causes increased phosphorylation of NPM on T199 and, to a lesser degree, S4. T199 phosphorylation is dependent on cdk2 but activators of cdk2 were not elevated. Upon inhibition of cdk2, NPMc-overexpressing cells demonstrate a greater G2/M phase arrest than wtNPM or GFP counterparts. However, the number of cells with 2 centrosomes did not increase concordantly. This suggests that the arrest was caused by a delay in centrosome duplication, most likely due to the inhibition of centrosome duplication caused by unphosphorylated NPMc. Overall, these results suggest that the phosphorylation of T199 is important in the mitotic progression of NPMc-expressing cells. This further supports the hypothesis that NPMc is associated with normal karyotypes in AML because the higher cytoplasmic load of NPM can better suppress centrosome overduplication which would otherwise result in unequal segregation of chromosomes during mitosis, leading to aneuploidy and other genomic instabilities.

Acute myeloid leukemia (AML) is a heterogeneous hematological malignancy whose symptoms include hemorrhages, weakness, anemia and susceptibility to infections^1^. Treatment involves leukapheresis, bone marrow transplantation and chemotherapy which is often prescribed based on the karyotype of the leukemic cells^2^.

Genomic instability has been described as one of the hallmarks of cancer^3^ and acute myeloid leukemia (AML) is no exception with 50–60% of cases having an abnormal karyotype. Such chromosomal abnormalities may be caused by improper segregation of chromosomes during mitosis^4^ or defects in DNA damage repair^5^,^6^. Of the 40–50% with normal karyotype, 61.7%^7^ heterozygously^8^ express a mutant form of nucleophosmin (NPMc)^9^ that is delocalized to the cytoplasm in contrast to the wild-type (wt) which is predominantly localized in the nucleolus^10^. Nucleolar localization is facilitated by a nucleolar localization signal at the extreme C-terminal end of the 294 amino acid protein which contains 2 tryptophans (W288 and 290) of which at least one is lost in the NPMc mutant^11^. In addition to this, a nuclear export signal is introduced and it is the synergistic effect of both these mutations that causes the aberrant dislocation of NPM to the cytoplasm^12^,^13^.

During the cell cycle, NPM is phosphorylated by cdk2-cyclin E on threonine 199 (T199) which causes the dissociation of NPM away from the centrosome and permits duplication. T199 phosphorylation likely continues through S and G2 phases via cdk2-cyclin A phosphorylation before NPM reassociates with the duplicated centrosomes during mitosis where it is phosphorylated by the mitotic cyclin B-cdk1 complex^14^ on T234 and T237^15^.

Mutation of T199 to non-phosphorylatable alanine prevents phosphorylation and hence centrosome duplication, thus increasing the number of cells with one centrosome and decreasing the number of cells with two centrosomes^16^. Without this phosphorylation, NPM associates specifically on unduplicated centrosomes and after phosphorylation dissociates leaving duplicated centrosomes free of NPM^17^.

As previous studies regarding centrosome duplication have pertained to wtNPM which is predominantly in the nucleolus, there is a gap in the literature about what would happen if more NPM were to be delocalized into the same subcellular compartment (cytoplasm) as the centrosome. Here we show that the increased cytoplasmic load of NPM leads to increased T199 phosphorylation. Cells overexpressing NPMc become more dependent on this elevated phosphorylation for mitotic progression than their wtNPM counterparts as inhibition of this phosphorylation results in a greater accumulation of cells with 4n DNA content (G2/M phase arrest). Centrosome numbers did not change significantly which is in discord with the change in cell cycle profile. This desynchronization of the centrosome and DNA duplication cycles show that T199 phosphorylation is especially important for cell cycle progression when NPM is mislocalized in cytoplasm.

## Results

### More NPMc is phosphorylated earlier in the cell cycle than wtNPM

**Figure 1:**
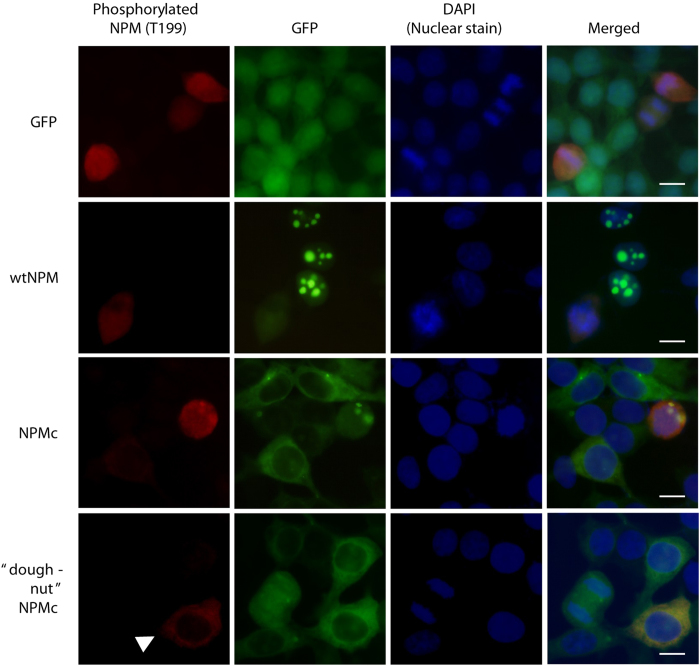
NPMc is phosphorylated earlier in the cell cycle than wtNPM. HEK293T stably expressing GFP, GFP-wtNPM (wtNPM) or GFP-NPMc (NPMc) were stained with rabbit anti-phosphoNPM(T199) followed by anti-rabbit Alexa fluor 568 conjugate. Unlike wtNPM and GFP expressing cells, immunofluorescence staining shows that some cells have phosphorylation of NPMc in the cytoplasm before nuclear membrane dissolution resulting in a “doughnut” shaped staining pattern around the nucleus (arrowhead). Bar = 20 μM.

In order to study the role of NPMcT199 phosphorylation in centrosome duplication, HEK293T cells were transduced to stably express green fluorescent protein (GFP), GFP tagged wtNPM (wtNPM) or GFP-NPMc (NPMc). Immunofluorescence staining of phosphorylated NPMT199 (NPMpT199) was apparent for all cell types (red, Fig. 1) in G2/M phase. The rounded shape, condensed DNA and whole cell staining of the NPMpT199^+^ cells indicates that these cells are in mitosis where the cell is detached from the culture plate and the nuclear envelope has broken down. In addition to these round mitotic cells, cells overexpressing NPMc uniquely display cytoplasmic staining of NPMpT199 in some cells that still have a semblance of their adherent form (Fig. 1, arrowhead; supplementary Fig. 1). The lack of staining in the nucleus of NPMcT199^+^ cells results in a doughnut-like staining pattern which is not seen in GFP or wtNPM-overexpressing cells. This is consistent with the hypothesis that in G1/S/G2 phases when the nuclear envelope is still intact and NPM is phosphorylated to permit centrosome duplication, many more molecules of NPM need to be phosphorylated in NPMc expressing cells due to the increased load of NPM in the cytoplasm.

**Figure 2:**
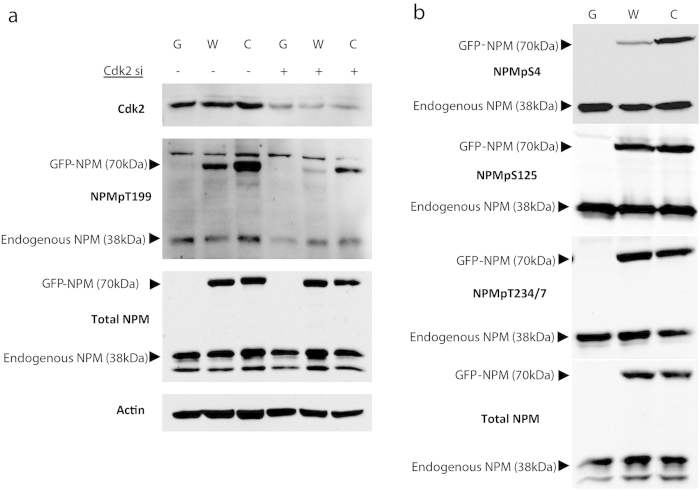
Cdk2-dependant NPMpT199 is hyperphosphorylated in NPMc and unique to pre-mitotic phosphorylation sites. (**a**) Western blot shows that knockdown of cdk2 reduces T199 phosphorylation and that NPMc phosphorylation is elevated compared to wtNPM. (**b**) Pre-mitotic phosphorylation site serine 4 (NPMpS4) also shows similar disparity between wtNPM and NPMc but there is no such difference for other centrosome related sites serine 125 (NPMpS125) or threonines 234 and 237 (NPMpT234/7). G, HEK293T stably overexpressing GFP; W, HEK293T stably overexpressing GFP-wtNPM; C, HEK293T stably overexpressing GFP-NPMc.

The earlier phosphorylation of NPMc is further reflected in western blot as elevated phosphorylation (Fig. 2a) since more NPM is phosphorylated for a longer period during the cell cycle. This elevated phosphorylation is not facilitated by increased cdk2 expression as cdk2 levels did not differ between GFP, wtNPM or NPMc overexpressing cells. Knockdown of cdk2 greatly reduced phosphorylation confirming previous literature that cdk2 is the predominant kinase for T199 phosphorylation^18^. Of the other centrosome related NPM phosphorylation sites, only serine 4 had a similar discrepancy as T199. Both phosphorylation of serine 125 which is associated with NPM homooligomerisation^19^ and threonines 234/237 (T234/7) which are associated with the centrosome during mitosis^15^ did not differ between wtNPM and NPMc (Fig. 2b). We propose that as the nuclear envelope breaks down during mitosis, this removes the relevancy of NPM’s cellular localisation to the phosphorylation of the mitotic T234/7 site because all cellular NPM regardless of whether it is wild-type or mutant is no longer physically separated from the centrosome resulting in similar phosphorylation levels. Serine 4 and T199, however, are phosphorylated pre-mitosis (S/G2 phase^20^) when the nuclear envelope separates wtNPM and the centrosome. In this case, the different subcellular locales of wtNPM versus NPMc become relevant in determining the level of pre-mitotic phosphorylation due to their different accessibilities to the centrosome. Indeed, relocalization of NPMc back into the nucleus using the export inhibitor leptomycin B reduces the phosphorylation of T199 (Supplementary Fig. 2 and 3).

### Increased phosphorylation is not dependent on cdk2 activation or localization

**Figure 3:**
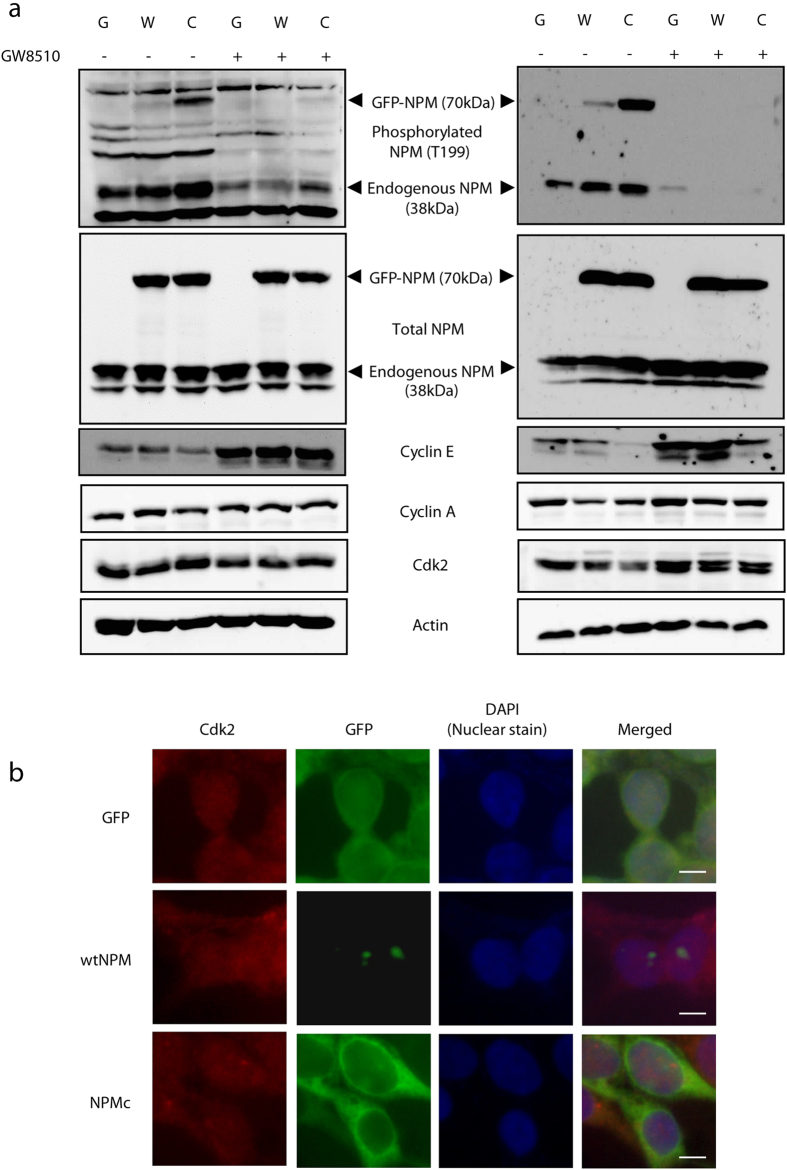
NPMc hyperphosphorylation is not dependent on cdk2 activation or localization. Western blot of selected cell cycle proteins in response to 5 μM GW8510 treatment for 24 hours shows cdk2 inhibition abolished the phosphorylation of both endogenous as well as GFP tagged exogenous NPM at threonine 199 (T199). (**a**) Left panel, HEK293T cells transfect with GFP (G), GFP-wtNPM (W) or GFP-NPMc (C) for 24 hours before drug treatment; Right panel, HEK293T stable cell lines stably overexpressing GFP (G), GFP-wtNPM (W) or GFP-NPMc (C). (**b**) Immunofluorescence staining of cdk2 indicates non-specific subcellular localization. Bar = 20 μM.

To further confirm the elevated phosphorylation of NPMc was not due to indirect effects of transgene insertion, HEK293T cells were transfected with GFP, GFP-wtNPM or GFP-NPMc and treated with cdk2 inhibitor^21^,^22^,^23^ GW8510. GFP-NPMc is more phosphorylated than GFP-wtNPM despite similar total NPM levels for both transfected HEK293T (left panel, Fig. 3a) as well as HEK293T stable cell lines (right panel, Fig. 3a). This phosphorylation was greatly diminished upon cdk2 inhibition across all cell types. Neither cyclin A or E which are both activators of cdk2 were elevated in GFP-NPMc overexpressing cells, indicating that the increased phosphorylation of NPMc does not require increased activation or protein levels of cdk2. In fact, cyclin E was expressed at lower levels in GFP-NPMc overexpressing cells despite the elevated phosphorylation of NPMc. Upon GW8510 treatment, cyclin E levels increased as would be expected because cdk2 is unable to stimulate the progression to mitosis and more cells become arrested and build up in S phase when cyclin E is expressed. Furthermore, immunofluorescence staining of cdk2 shows that it is not restricted to any subcellular location, neither does its localization differ between wild-type or NPMc-overexpressing cells (Fig. 3b). The elevated phosphorylation of NPMc is therefore more likely due to the increased cytoplasmic load of NPM rather than the activation or localization of cdk2.

### Phosphorylation inhibition causes greater G2/M arrest of cells with only one centrosome in NPMc-overexpressing cells than wtNPM and GFP counterparts

**Figure 4:**
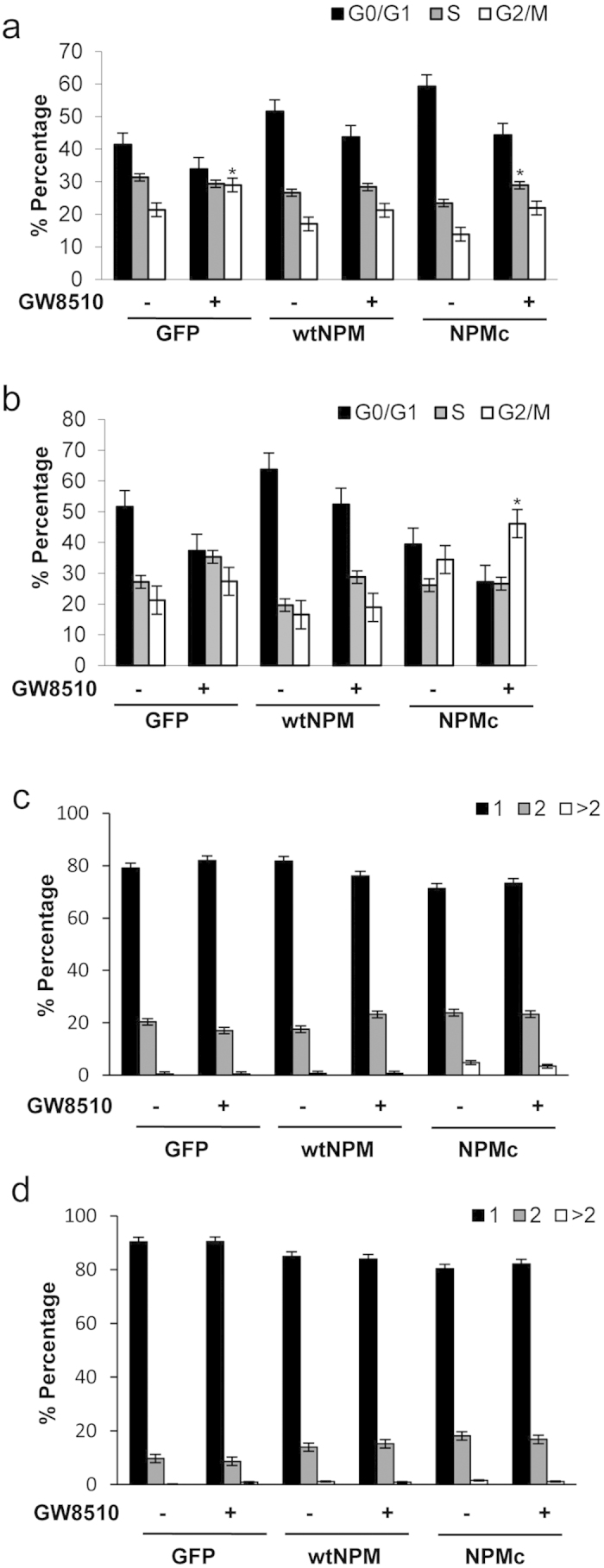
NPMc-overexpressing cells have greater increase in 4n cells than wtNPM- or GFP-expressing counterparts with no change in centrosome numbers. Cells were treated as per Fig. 3 and DNA was stained with potassium iodide and measured using fluorescence-associated cell sorting (FACS). (**a**) Cell cycle analysis of non-synchronized cells. Statistical significance was calculated using student’s T-test in comparison to DMSO controls. (**b**) Cell cycle analysis of synchronized cells. Cells were synchronized by serum starvation for 72 hours and then released into complete media containing GW8510 or the control solvent dimethyl sulfoxide (DMSO) for 24 hours. Statistical significance was calculated using student’s T-test in comparison to DMSO controls. Centrosome numbers in non-synchronized cells (**c**) and synchronized cells (**d**). Cells were treated with or without cdk2 inhibitor GW8510 and then fixed and stained for γ-tubulin protein which is specific to centrosomes (supplementary Fig. 4). 1, cells with 1 centrosome; 2, cells with 2 centrosomes; >2, cells with 3 or more centrosomes. Results show mean and standard error from three independent experiments.

As western blot showed that NPMc had greater T199 phosphorylation than wtNPM, we hypothesized that cells overexpressing NPMc are more reliant on this phosphorylation for cell cycle progression. Thus, we reduced T199 phosphorylation using the cdk2 inhibitor drug GW8510 as the process of transfecting siRNA in itself caused complicating changes to the cell cycle which we wanted to avoid. As shown in Fig. 3a, GW8510 effectively reduced T199 phosphorylation. Upon treatment with GW8510, all cells showed a decrease in the number of 2n cells (G0/G1 phase) and a corresponding increase in the number of cells with 4n DNA content (G2/M, Fig. 4a,b). GFP-NPMc expressing cells showed the greatest G2/M phase arrest for both non-synchronized (Fig. 4a) and synchronized cells (Fig. 4b). For non-synchronized cells, GW8510 treatment of GFP-NPMc overexpressing cells had an increase of 8.1% points from 13.9% of G2/M phase cells to 22.0%; compared to the GFP overexpressing control which increased 7.6% points from 21.4% to 29.0% and GFP-wtNPM which increased only 4.3% from 17.0% to 21.3%. However, the increase of 4n cells in NPMc-overexpressing cells was not statistically significant which could be due to the fact that in the non-synchronized system, cells reach the cell cycle stage where cdk2 activity is needed at different times and at the point of harvesting, only some cells have reached the “roadblock” created by GW8510’s cdk2 inhibition. Therefore, we repeated the drug treatment with synchronized cells so that most of the cells would reach the “roadblock” created by GW8510 at the same time. Indeed in synchronized cells, the greater G2/M phase arrest of GW8510-treated GFP-NPMc overexpressing cells was more apparent, with the number of G2/M phase cells exceeding those in G0/G1 (Fig. 4b). This demonstrates a greater reliance of NPMc expressing cells on cdk2 activity for mitotic progression.

Despite the obvious difference in cell cycle response to cdk2 inhibition, centrosome numbers did not change significantly for both synchronized and non-synchronized cells (Fig. 4c,d) indicating that the centrosome and DNA replication cycle were decoupled. The increase in 4n cells without a concurrent increase in cells with 2 centrosomes upon cdk2 inhibition suggests that the DNA has completed its duplication but not the centrosome thus preventing mitotic progression causing the accumulation of cells in G2/M phase. As NPMc-overexpressing cells have the greatest number of arrested cells, this suggests that cells with NPMc have a greater dependency than wtNPM on cdk2 activity and T199 phosphorylation for centrosome duplication and mitotic progression.

### Alanine-mutated NPMc mimics G2/M phase arrest caused by cdk2 inhibition

**Figure 5:**
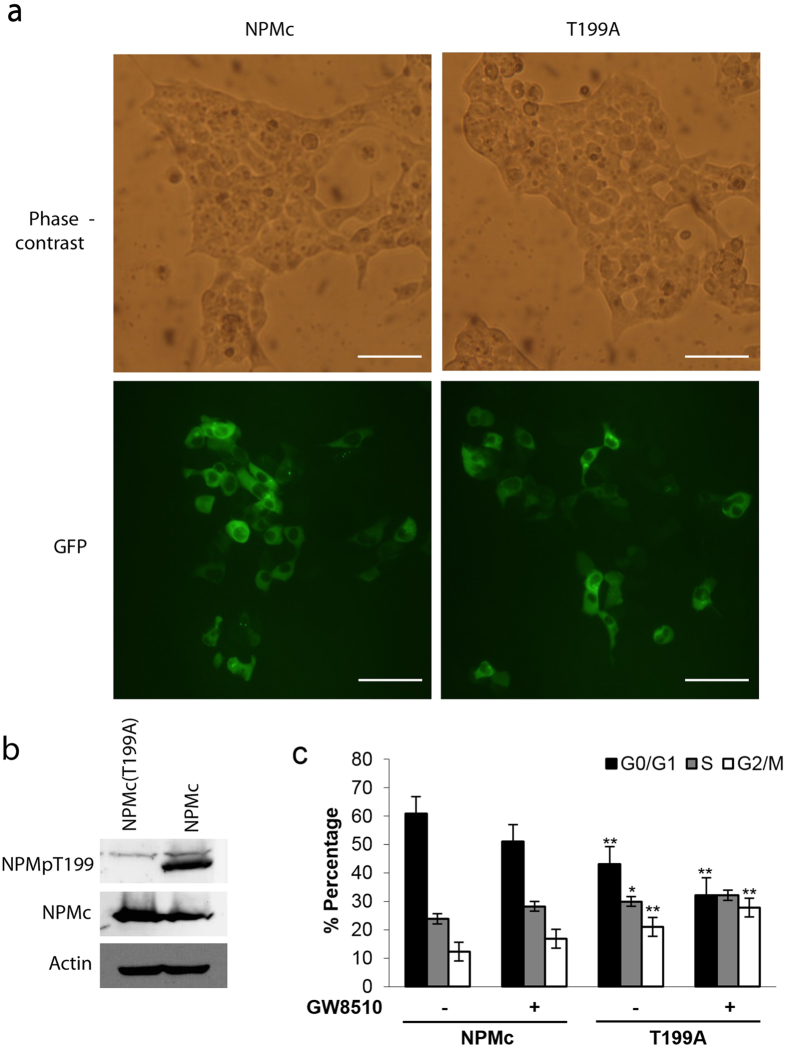
Alanine-mutated NPMc mimics G2/M phase arrest caused by cdk2 inhibition. (**a**) Light microscope images of HEK293T transfected with GFP-NPMc (NPMc) or GFP-NPMc with mutated T199A (T199A). Both (NPMc) (T199A) have the same subcellular localization (cytoplasm). Bar = 100 μM. (**b**) Western blot shows that NPMcT199A is not detectable by antibody specific for phosphorylated NPM (NPMpT199). (**c**) HEK293T were transfected with GFP-NPMc (NPMc) or GFP-NPMc with mutated T199A (T199A) and treated with GW8510 or the solvent DMSO as per Fig. 3 24 hours post-transfection. Results show mean and standard error from three independent experiments. *p-value < 0.05, **p-value < 0.01. Statistical significance was calculated using student’s T-test compared to the respective NPMc treatment. G0/G1, cells with 2n DNA content; S, cells synthesizing DNA; G2/M, cells with 4n DNA content.

To show that G2/M phase arrest was not due to unspecific effects of the drug, site-directed mutagenesis was used to change T199 to non-phosphorylatable alanine. HEK293T were then transfected with either GFP-NPMc or the mutated GFP-NPMcT199A and treated with GW8510 before cell cycle analysis using FACS. Western blot shows that the T199A mutant is undetectable by the phospho-T199 antibody (Fig. 5b) and the localization of the T199A mutant was cytoplasmic showing that the mutation did not affect the localization of GFP-NPMc (Fig. 5a). This inhibition of NPMcT199 phosphorylation by site-directed mutagenesis caused a cell cycle arrest similar to drug treatment indicating that the increase in G2/M phase cells is not due to any indirect effect of GW8510 (Fig. 5c).

### NPMc expressing AML cell line also has elevated T199 phosphorylation

**Figure 6:**
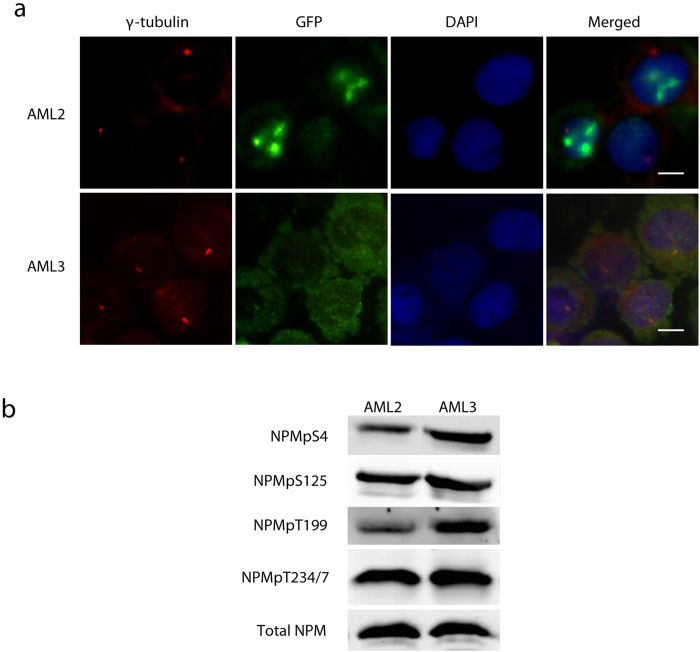
T199 is likewise hyperphosphorylated in NPMc-expressing cell line OCI-AML3. (**a**) Immunofluorescence co-staining of centrosome marker γ-tubulin (red puncta) and NPM (green) show that OCI-AML3 (AML3) has cytoplasmic NPM while OCI-AML2 (AML2) has wtNPM which is localised in the nucleus and separated from the centrosome by the nuclear envelope. Bar = 10 μM. (**b**) Western blot of selected NPM phosphorylation sites. T199 is more phosphorylated in AML3 compared to AML2 whereas S4 phosphorylation is moderately elevated in AML3. Other phosphorylation sites are not significantly different.

OCI-AML3 (AML3) is the only AML cell line that has so far been found to heterozygously express the NPMc mutation^8^. Immunofluorescence staining of NPM in AML3 shows that NPM is cytoplasmically localised in contrast with another AML cell line (OCI-AML2) which bears nuclear localized wtNPM^24^ (Fig. 6a). Western blot of selected centrosome phosphorylation sites show that while S125 and T234/7 were not disparately phosphorylated, the pre-mitotic phosphorylation sites T199 and S4 have higher phosphorylation in AML3 compared to AML2, with T199 having the greatest disparity (Fig. 6b). This further supports the importance of NPM’s accessibility to the centrosome which is restricted by the nuclear envelope in pre-mitotic wtNPM-expressing cells. By contrast, we posit that in NPMc-expressing AML3, NPMc’s ready access to the centrosome leads to compensatory hyperphosphorylation to prevent too much NPM binding to the centrosome which would inhibition centrosome duplication and cell proliferation.

## Discussion

Previous studies on NPM and centrosomes have focused on wtNPM which is predominantly localized in the nucleus, meaning that only a small fraction of total cellular NPM is associated with the centrosome in the cytoplasm. Here we show that in NPMc—overexpressing cells, increased phosphorylation is needed to compensate for the elevated cytoplasmic load of NPM. Therefore at the G1/S phase transition when the nuclear envelope is not yet dissolved and NPM needs to be phosphorylated for centrosome duplication, much more NPM is phosphorylated in NPMc-overexpressing cells (Figs. 2a and 3a). We also show that like wtNPM^16^,^18^,^25^, the phosphorylation of NPMc primarily dependent on cdk2. When this is perturbed, cells accumulate pre-mitosis with duplicated 4n DNA (Fig. 4a,b) but only 1 centrosome (Fig. 4c,d) instead of the normal 2. The decoupling of the DNA and centrosome cycle upon cdk2 inhibition and decreased NPM phosphorylation shows that while NPM phosphorylation may be dispensable for cell cycle progression in wtNPM^26^ this is not the case for cytoplasmic NPM. Future experiments would be needed to strengthen this theory by eliminating other potential inhibitors of mitotic progression e.g. DNA damage, faulty microtubule machinery. In addition, NPMc has recently been shown to relocalize into the nucleolus using the drug avrainillamide^27^ and it would be interesting to see if such relocalization reduces the need for NPM phosphorylation for centrosome duplication and mitotic progression.

While this study shows that the elevated phosphorylation is not dependent on cdk2 activity or localization, the exact mechanism remains to be elucidated. It may be that NPMc’s C-terminal mutation could alter its binding affinity for cdk2. However, previous studies of NPMc’s role in AML show that it’s interaction with binding partners does not change, but rather it is the aberrant localization these binding partners with NPMc into the cytoplasm that contribute to leukemogenesis^28^,^29^,^30^,^31^,^32^. Future in vitro work would shed light on the kinetics of NPMc/cdk2 phosphorylation.

As seen in Fig. 2, serine 4 is also similarly hyperphosphorylated in NPMc compared to wtNPM. Historically, it is less well researched than T199. For example, there is no commercially available antibody for NPMpS4 that is suitable for immunofluorescence. In addition, unlike T199 which is predominantly phosphorylated by cdk2, the study of NPMpS4 is compounded by the fact that it is phosphorylated at least 2 different kinases at different stages throughout the cell cycle^33^. Regardless, this remains an interesting avenue to pursue as it has been suggested that there is some functional relationship between T199 and S4^20^.

Cyclin E/cdk2 is the primary kinase of NPMpT199. However, it is known that there is a certain level of redundancy among the cyclins/Cdks^34^ and other Cdks may be able to phosphorylate NPM and still allow centrosome duplication to proceed. However, western blot (Fig. 3a) shows that NPM is significantly reduced upon cdk2 inhibitor GW8510 as well as cdk2 knockdown, implying that there is little compensatory phosphorylation for NPMpT199, at least in HEK293T. Another possibility is that given NPM is a multifunctional protein and has multiple binding partners, these other proteins may be able to somehow chaperone NPM away from the centrosome and allow duplication to initiate. Whatever the case, it is clear that when cdk2 functionality and T199 phosphorylation is compromised, any alternative attempt to displace NPM from the centrosome is not as efficient, especially when the cytoplasmic load of NPM is increased as in NPMc-overexpressing cells, causing an arrest of 4n pre-mitotic cells more so than in wtNPM or GFP counterparts (Fig. 4a,b). Future work into other Cdks and interactions with other centrosome proteins such as BRCA2^35^ will be needed to further expound the effects of NPMc in cell cycle and centrosome duplication.

In conclusion, we demonstrate that the increased cytoplasmic load of NPMc increases the reliance on cdk2-based T199 phosphorylation for cell cycle progression. As ~85% of NPMc+ AML have a normal karyotype^10^, we propose that NPMc has a role to play in centrosome duplication as the increased cytoplasmic load of NPM can prevent centrosome reduplication thereby maintaining the normal karyotype. We have also shown that serine 4 is similarly disparately phosphorylated^34^, so future studies into how both these sites could also affect centrosome duplication and the cell cycle will provide new potential targets in the treatment of NPMc+ AML.

## Methods

### Stable cell lines

Stable cell lines were generated by transduction of the gene of interest cloned into the pLenti expression vector using the ViraPower^TM^ HiPerform Expression Kit (Invitrogen) following the manufacturer’s recommendations.

GFP, GFP-wtNPM and GFP-NPMc were amplified from previously constructed pAcGFP plasmids using forward primer of sequence 5′- TAG TGA ACC GTC AGA TCC GC -3′ and reverse primer 5′- TGA TCA GTT ATC TAG ATC CGG TGG -3′ and ligated to pLenti6.3/V5-TOPO following the manufacturer’s recommendations. HEK293FT cells were transfected with 9 μg of packaging mix and 2 μg of pLenti vector construct using 25 μl of Lipofectamine^TM^ 2000 (Invitrogen). The DNA and Lipofectamine^TM^ 2000 were incubated in 3 ml of serum-free Opti-MEM (Sigma) for 20 mins before addition to a 10 cm plate of HEK293FT cells with 5 ml of Opti-MEM media containing 10% FBS. Following overnight incubation, cells were checked for fluorescence and the media was changed to complete DMEM without G418. Media was collected the following day and centrifuged at 2500 g for 15 mins at room temperature to remove cell debris. Polybrene (8 μg/ml) was then added to the supernatant and then the supernatant was used to resuspend cell pellets of HEK293T which had been harvested from one 10 cm plate of confluent cells by centrifugation at 2000 rpm for 5 mins. The cells were then centrifuged at 900 g for 1 hour at room temperature and then resuspended using the same supernatant and plated. The next day, the transduction process was repeated using fresh supernatant from the HEK293FT producer cell line. Virus-containing media was replaced the next day with RPMI. Two days later, the cells were checked for fluorescence and blasticidine (0.6 μg/ml) was added. Media with blasticidine was replenished every 3-4 days for 2-3 weeks.

### Cell culture and transfection

HEK293T were cultured in RPMI 1640 (Sigma) supplemented with 10% fetal bovine serum and 1% penicillin streptomycin. Cells were maintained in a 37 °C incubator with a humidified atmosphere of 5% CO_2_ and passaged at 90% confluence.

NPM genes had been cloned into pAcGFP-C1 plasmids (Clontech). 5 μg of plasmid was transfected with 10 μl of Lipofectamine 2000 (Invitrogen) according to the manufacturer’s instructions. For cdk2 knockdown, 100 nM of cdk2 siRNA (Santa Cruz) was transfected with 10 μl of Dharmafect (GE healthcare).

### Immunofluorescence

HEK293T cells were grown on glass cover slips while hematopoietic cell lines OCI-AML2 and OCI-AML3 were spun down onto glass slides at 600 rpm for 5 min. For NPMpT199 staining, cells were fixed with 4% paraformaldehyde in phosphate-buffered saline (PBS) for 30 min, followed by a wash with PBS and 5 min permeabilization in digitonin (50 μg/ml in PBS) and quenched for 5 min with 50 mM NH_4_Cl in PBS. For γ-tubulin or cdk2 staining, cells were fixed/permeabilised in 50:50 ice-cold acetone:methanol for 30 min and then washed once with PBS before proceeding to blocking. Blocking buffer consists of 5% goat serum and 2% horse serum in PBS for 1 hour at room temperature. Samples were incubated with γ-tubulin primary antibody (T3559, Sigma) at 1:300 dilution factor, cdk2 primary antibody (sc-748, Santa Cruz) at 1:50 dilution factor or phospho-NPM^T199^ primary antibody (3541, Cell signaling) at 1:500 dilution factor in blocking buffer at room temperature for 1 hour. Alexa Fluor-conjugated anti-mouse or anti-rabbit immunoglobulins (Life Technologies) were used as secondary antibodies (1:1000 in blocking buffer for 1 hour at room temperature) and cell nuclei were visualized with DAPI. Fluorescent images were taken using BX60 microscope (Olympus) and processed using Image J. 100 cells were counted per experiment for centrosome counting. Experiments were conducted in triplicate and two-sample unequal variance T-test was used to calculate p-value.

### Western blotting

Cells were washed once with PBS and resuspended in 100 μl of hot SDS lysis buffer (100 mM Tris-HCL pH8.0, 2% sodium dodecyl sulfate, 50 mM dithiothreitol, 20% glycerol) and incubated at 85 °C for 10 mins. Samples were then sonicated at 40% amplitude for 15 s in 1 s pulses with 1 s of pause between each pulse followed by another 10 mins incubation at 85 °C. Protein concentration was estimated by measuring absorbance at 280 nm using the Thermo Scientific NanoDrop 2000™ Spectrophotometer. Proteins were separated under denaturing conditions in 12% polyacrylamide gels and electroblotted onto nitrocellulose membranes using Trans-Blot SD semi-dry transfer (1620112, Bio-Rad) at 20 V for 35 min. Membranes were blocked with 3% w/v skimmed milk in Tris buffered saline with 0.1% v/v Tween-20 (TBST) for 15 mins followed by overnight incubation with primary antibodies diluted in 5% w/v bovine serum albumin in TBS. Blots were then washed in TBST 3 times for 5 mins each time before a 1 hour incubation at room temperature with horse-radish peroxidase (HRP) conjugated goat anti-mouse or anti-rabbit secondary antibodies (Santa Cruz Biotechnology) in blocking buffer. Protein bands were visualized with Pierce Biotechnology SuperSignal West Pico or Femto chemiluminescent substrate following the manufacturer’s recommendations.

### Cell synchronisation

Cells were starved for 72 hours in serum-free media and then released into complete media with 5μM GW8510 (sc-215122, Santa Cruz) or the solvent DMSO for 24 hours.

### Cell cycle analysis

Cells were fixed in 1 ml of ice-cold 70% ethanol for at least 3 hours then washed once with PBS and resuspended in Millipore Muse cell cycle reagent (MCH100106, Merck Millipore) as per the manufacturer’s instructions. 10,000 events per sample was measured in the Muse Cell Analyzer (0500-3115, Merck Millipore). Experiments were conducted in triplicate and two-sample unequal variance T-test was used to calculate p-value.

## Additional Information

**How to cite this article**: Chan, N. and Meng Lim, T. Cytoplasmic nucleophosmin has elevated T199 phosphorylation upon which G2/M phase progression is dependent. *Sci. Rep.* **5**, 11777; doi: 10.1038/srep11777 (2015).

## Supplementary Material

Supplementary Information
