# Supplementary material for: Cytoplasmic nucleophosmin has elevated T199 phosphorylation upon which G2/M phase progression is dependent
Source: Sci Rep. 2015 Jun 30;5:11777. doi: 10.1038/srep11777 (PMC4485321; doi:10.1038/srep11777)
Supplement: Supplementary Information [file srep11777-s1.pdf]

Cytoplasmic nucleophosmin has elevated T199 phosphorylation upon which G2/M phase progression is dependent.

Narisa Chan, Tit Meng Lim[1]

Department of Biological Sciences, National University of Singapore, 14 Science Drive 4, Singapore 117543.

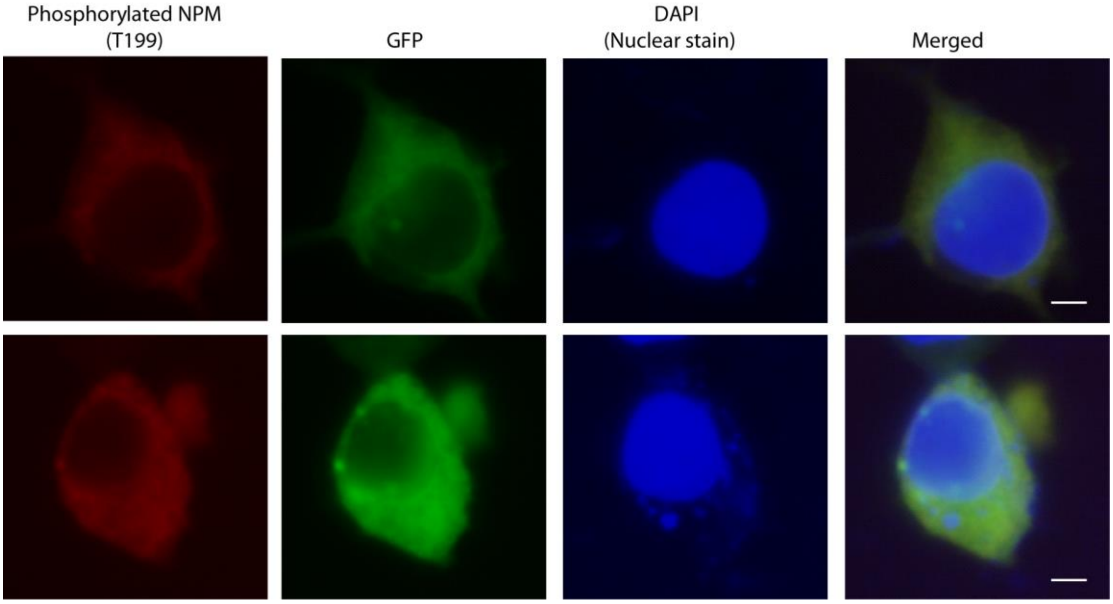

Supplementary Figure 1. Further examples of "doughnut" NPMpT199 staining in GFP-NPMc overexpressing HEK293T.
Immunofluorescence staining of phosphorylated NPM (red) which is excluded from the nucleus by the nuclear envelope and overlaps with GFP signal from GFP-tagged NPMc. Bar=10μM.

[1] Correspondence to: dbsltm@nus.edu.sg

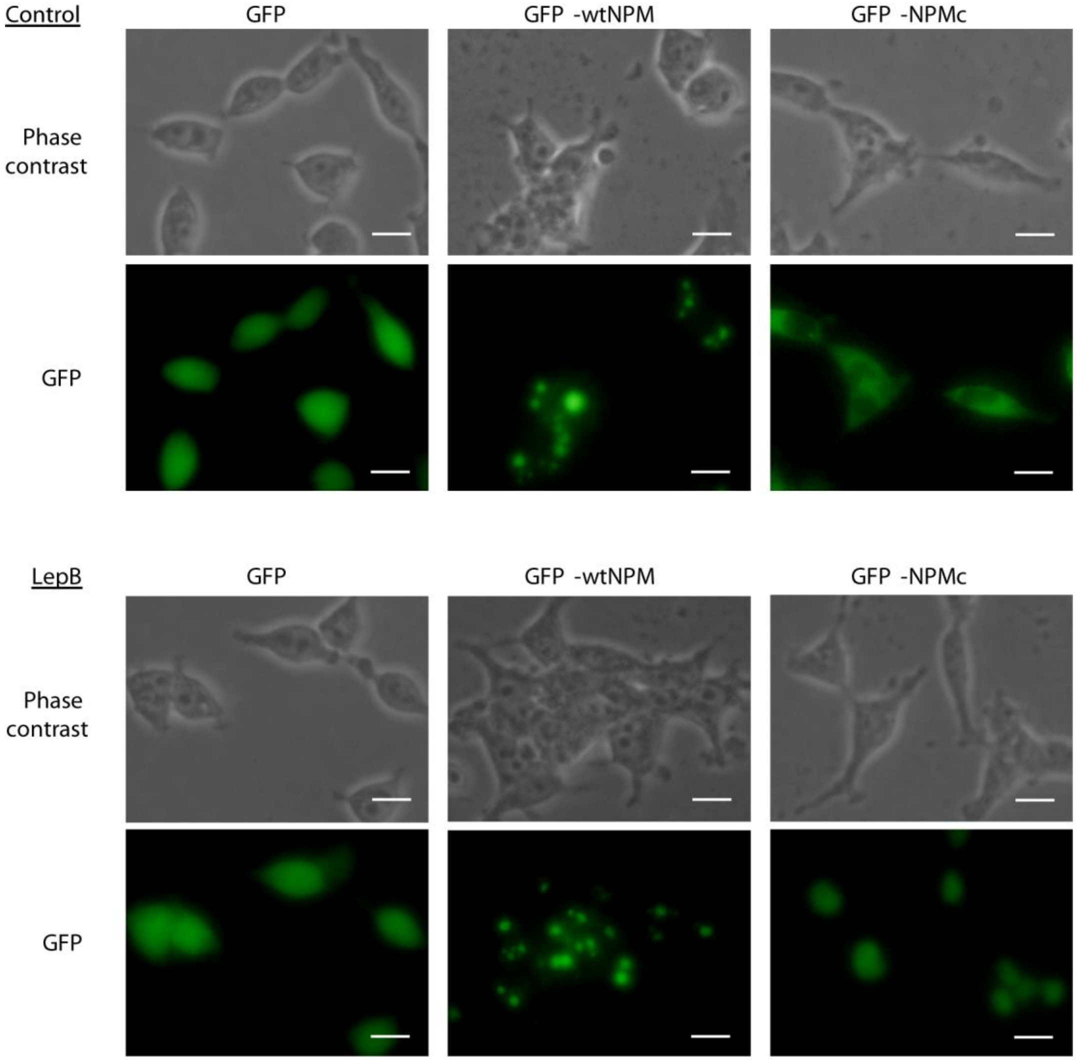

Supplementary Figure 2. Leptomycin B causes relocalisation on NPMc into the nucleus but does not affect wtNPM or GFP localization.
HEK293T stably expressing GFP, GFP-wtNPM (wtNPM) or GFP-NPMc (NPMc) were treated with 5nM leptomycin B (lepB, bottom 6 panels) for 24 hours. The localisation of NPMc (bottom right most panel) changes from cytoplasmic to nuclear as is consistent with the nuclear export inhibitor action of the drug. GFP and GFP-wtNPM do not show any visible change in subcellular localisation. Bar=30μM.

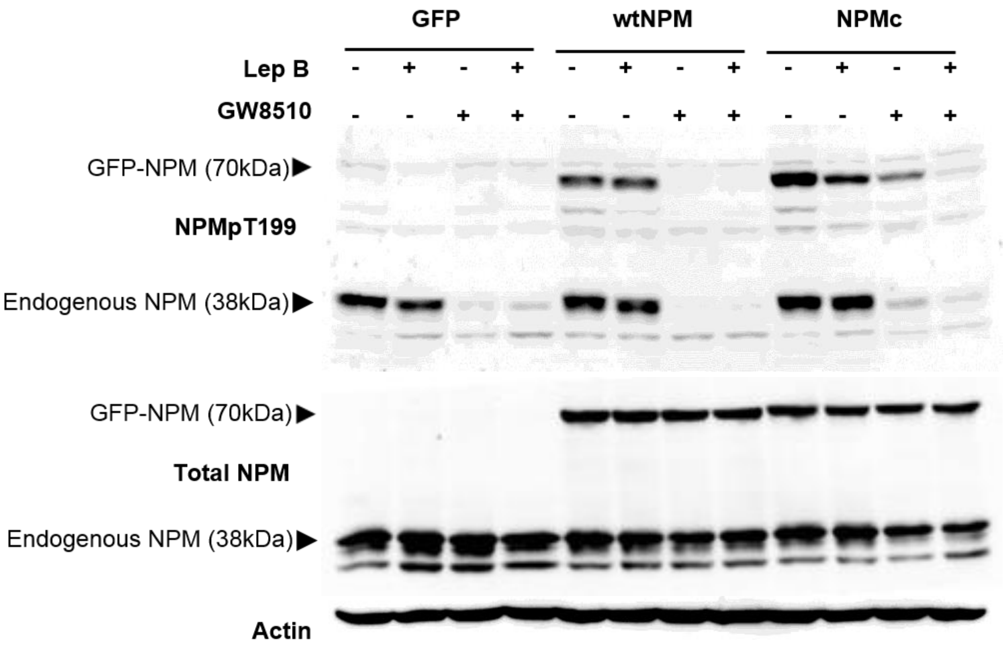

Supplementary Figure 3. Leptomycin B reduces NPMc's T199 phosphorylation but not that of wtNPM.

Cells were treated with leptomycin B as per figure 2 and western blotted. Treatment with leptomycin B alone reduced T199 phosphorylation of NPMc but not wtNPM whereas cdk2 inhibitor, GW8510 (5μM, 24 hrs) affected both wtNPM and NPMc. Co-treatment of leptomycin B with GW8510 further reduced the phosphorylation of NPMcT199. Lep B, leptomycin B; GW8510, cdk2 inhibitor.

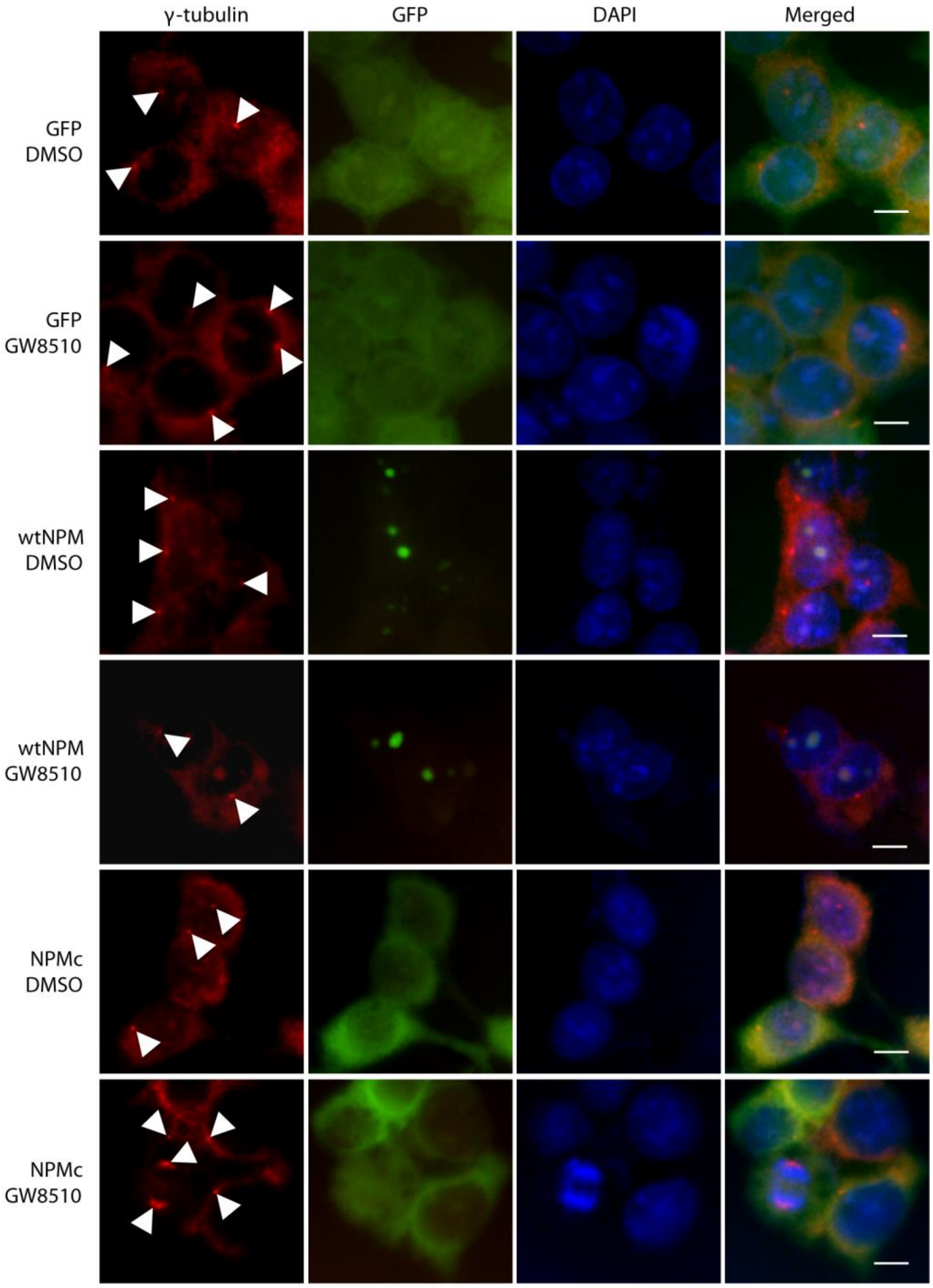

Supplementary Figure 4. Examples of centrosome staining in HEK293T stable cell lines under control (DMSO) or GW8510 treatments.

Centrosomes appear as distinct red puncta (arrowheads) which can be counted. HEK293T stably expressing GFP, GFP-wtNPM (wtNPM) or GFP-NPMc (NPMc) were stained with rabbit anti-γ-tubulin followed by anti-rabbit Alexa fluor 568 conjugate. Bar=20μM. DMSO, cells treated 0.1% v/v of the solvent dimethyl sulfoxide as control for 24 hours; GW8510, cells treated with 5μM of the cdk2 inhibitor for 24 hours; GFP, HEK293T stably overexpressing GFP; wtNPM, HEK293T stably overexpressing GFP-wtNPM; NPMc, HEK293T stably overexpressing GFP-NPMc.
